# Maternal High Fat Diet Alters Skeletal Muscle Mitochondrial Catalytic Activity in Adult Male Rat Offspring

**DOI:** 10.3389/fphys.2016.00546

**Published:** 2016-11-18

**Authors:** Chantal A. Pileggi, Christopher P. Hedges, Stephanie A. Segovia, James F. Markworth, Brenan R. Durainayagam, Clint Gray, Xiaoyuan D. Zhang, Matthew P. G. Barnett, Mark H. Vickers, Anthony J. R. Hickey, Clare M. Reynolds, David Cameron-Smith

**Affiliations:** ^1^Liggins Institute, University of AucklandAuckland, New Zealand; ^2^College of Sport and Exercise Science, Institute of Sport, Exercise and Active Living, Victoria UniversityMelbourne, VIC, Australia; ^3^Applied Surgery and Metabolism Laboratory, School of Biological Sciences, University of AucklandAuckland, New Zealand; ^4^Gravida: National Centre for Growth and Development, University of AucklandAuckland, New Zealand; ^5^Food Nutrition and Health Team, Food and Bio-based Products Group, AgResearch GrasslandsPalmerston North, New Zealand

**Keywords:** skeletal muscle, mitochondria, maternal high fat, developmental programming

## Abstract

A maternal high-fat (HF) diet during pregnancy can lead to metabolic compromise, such as insulin resistance in adult offspring. Skeletal muscle mitochondrial dysfunction is one mechanism contributing to metabolic impairments in insulin resistant states. Therefore, the present study aimed to investigate whether mitochondrial dysfunction is evident in metabolically compromised offspring born to HF-fed dams. Sprague-Dawley dams were randomly assigned to receive a purified control diet (CD; 10% kcal from fat) or a high fat diet (HFD; 45% kcal from fat) for 10 days prior to mating, throughout pregnancy and during lactation. From weaning, all male offspring received a standard chow diet and soleus muscle was collected at day 150. Expression of the mitochondrial transcription factors nuclear respiratory factor-1 (NRF1) and mitochondrial transcription factor A (mtTFA) were downregulated in HF offspring. Furthermore, genes encoding the mitochondrial electron transport system (ETS) respiratory complex subunits were suppressed in HF offspring. Moreover, protein expression of the complex I subunit, NDUFB8, was downregulated in HF offspring (36%), which was paralleled by decreased maximal catalytic linked activity of complex I and III (40%). Together, these results indicate that exposure to a maternal HF diet during development may elicit lifelong mitochondrial alterations in offspring skeletal muscle.

## Introduction

Significant increases in type 2 diabetes (T2D) and obesity rates that have been observed worldwide over the past three decades are the combined result of high energy intake, a sedentary lifestyle and genetic predisposition (Hill and Peters, 1998). However, there is also evidence of inter-generational programming of metabolic alterations that may predispose subsequent generations to an increased risk of developing metabolic disease (Barker, 2007). Therefore, an adverse intrauterine and/or early life nutritional environment may have lifelong consequences for the metabolic status of the offspring (Godfrey and Barker, 2001; Godfrey et al., 2010). High maternal energy intake predisposes offspring to hyperphagia, increased adiposity, hypertension, blunted systemic glucose disposal, and chronic inflammation in adulthood (Bayol et al., 2005; Samuelsson et al., 2008; Pileggi et al., 2015). Although it is well established that a maternal high-fat diet leads to hyperinsulinemia in offspring (Howie et al., 2009, 2013), the skeletal muscle molecular defects that contribute to poor metabolic outcomes remain poorly elucidated.

Alterations in the structure and functional oxidative capacity of skeletal muscle mitochondria that lead to disturbances in mitochondrial respiration are hallmark features in the development of insulin resistance and T2D (Kelley et al., 2002; Mogensen et al., 2007; Patti and Corvera, 2010). The mitochondrial electron transport system (ETS) transfers electrons through complexes I–IV to generate the membrane potential that drives ATP formation, a process collectively named oxidative phosphorylation (OXPHOS). The expression of genes that encode the ETS complex subunits are subject to transcriptional regulation by the transcription co-activator proliferator-activated receptor γ coactivator α (PGC1α), and the nuclear transcription factor nuclear regulatory factor 1 (NRF1). PGC1α and NRF1 are downregulated in muscle from individuals with T2D (Mootha et al., 2003; Patti et al., 2003). NRF1 also regulates expression of mitochondrial transcription factor A (mtTFA), which singularly controls the transcription of the mitochondrial genome (Virbasius and Scarpulla, 1994). Data from studies utilizing gene array technologies have identified decreased gene expression of critical subunits of the mitochondrial ETS respiratory complexes in skeletal muscle from human T2D subjects and subjects with a family history of T2D (Mootha et al., 2003; Patti et al., 2003). Moreover, preliminary evidence from rodent and human studies indicates that mitochondrial dysfunction may be programmed by the maternal environment (Taylor et al., 2005; Burgueño et al., 2013; Latouche et al., 2014). High maternal fat intake leads to insulin resistance in offspring (Armitage et al., 2005) which is associated with decreases in mitochondrial function and content including; liver mtDNA copy number (Taylor et al., 2005; Burgueño et al., 2013) aortic smooth muscle mitochondrial gene expression (Taylor et al., 2005), and activity of the ETS enzyme complex in liver (Bruce et al., 2009a). Furthermore, a maternal diet high in fat and sucrose resulted in the significant alteration of gene expression in the skeletal muscle transcriptome, including downregulation of genes associated with mitochondrial oxidative function (Latouche et al., 2014). However, to date, studies examining the effects of maternal diet on offspring skeletal muscle mitochondrial function have used mixed obesogenic maternal diets (Shelley et al., 2009; Latouche et al., 2014), leaving the effects specifically attributable to increased maternal saturated fat intake poorly defined.

Currently there is limited understanding of the complex mitochondrial changes that follow a maternal HF diet, as there are multiple signaling factors that regulate skeletal muscle mitochondrial function. Therefore, the aim of the present study was to quantify mitochondrial function in the skeletal muscle from male rat offspring exposed to a high-fat (HF) diet during pregnancy and lactation. We hypothesized that male offspring born to HF dams would exhibit decreased expression of genes encoding mitochondrial proteins, which would impair skeletal muscle mitochondrial function. Analysis was therefore undertaken to determine the expression of transcription factors known to modulate mitochondrial gene expression with complementary analysis of the genes that comprise the mitochondrial ETS respiratory complexes. Following this, *ex-vivo* maximal enzymatic activity of the mitochondrial respiratory complexes were spectrophotometrically examined (Spinazzi et al., 2012). Additionally, skeletal muscle fiber type was determined by immunofluorescence to assess the muscular phenotype.

## Methods

### Animal experiments

Animal protocols for this study have previously been described (Segovia et al., 2015). In brief, female Sprague-Dawley rats (100 days of age) were housed under standard conditions at 25°C with 12 h light/dark cycle (7.00 am–7.00 pm) with *ad libitum* access to food and water. Rats (*n* = 12) were randomly assigned to receive either (1) a purified control diet (CD *n* = 6, 10% kcal from fat; Research Diets Inc., NJ) or (2) a purified high fat diet (HF *n* = 6; 45% of kcals from fat from lard, Research Diets, D12451), for 10 days prior to mating and throughout pregnancy and lactation. Following the pre-gestational feeding period, female rats were time-mated using an estrus cycle monitor (EC40, Fine Science Tools, Foster City, CA). Day 1 of pregnancy was determined by detection of spermatozoa by vaginal lavage, and dams were individually housed. To ensure standardized nutrition until weaning, litters were adjusted to 8 pups (4 male, 4 female) at postnatal day 2. At weaning (day 21), male siblings from each litter were housed two per cage and maintained *ad libitum* on a standard chow diet (Diet, 2018; Harlan Teklad, Oxo, UK) for the remainder of the study (day 150). A minimum of 5 litters were assessed per maternal dietary group; female offspring were not assessed in the present study due to confounds caused by the female estrus cycle. All animal work was approved by the Animal Ethics Committee (Approval R1069) at The University of Auckland.

### Tissue collection and preparation

Male offspring were fasted overnight prior to tissue collection at day 150. Animals were decapitated following an intraperitoneal injection of sodium pentobarbitone (60 mg/kg). The soleus muscle was excised from the right hindlimb, weighed, snap-frozen in liquid nitrogen and stored at −80°C for later analyses.

### Immunohistochemistry

Sections (2 cm) were cut mid-belly perpendicular to the muscle fiber axis from snap frozen soleus muscle. Samples were mounted in optimal cutting temperature compound and sectioned (10 μm) at −20°C using a cryotome (Leica Microsystems CM1850, Leica Biosystems Nussloch GmbH, Nussloch, Germany) for determination of muscle fiber type and size. Quantitative analysis for muscle fiber type (percentage of total fibers), and cross sectional area (CSA) were determined as previously described (Figueiredo et al., 2015). Air-dried sections were blocked in 10% goat serum for 1 h at room temperature and then immunolabelled overnight at 4°C with antibodies from the Developmental Studies Hybridoma Bank (University of Iowa, Iowa, IA) against Myosin heavy chain (MHC)-slow (BA-F8, IgG2b diluted 1:12.5), MHCIIa (SC-71, IgG1, 1:600), MHCIIb (BF-F3, IgM, 1:25), and dystrophin (MANDYS1[3B7], IgG2a, diluted 1:50) to stain for the basement membrane of muscle fibers. Sections were immunolabelled with Alexa Fluor® fluorescently conjugated Ig subclass specific Goat Anti-Mouse secondary antibodies (Alexa Fluor® 488 IgG1, Alexa Fluor® 350 IgG2b, Alexa Fluor® 555 IgM, Alexa Fluor® 647 IgG2a, all 1:500) for 1 h at room temperature. Mounted slides were visualized using a fluorescence microscope at 10x magnification (Axio Imager Z2, Carl Zeiss, Oberkochen, Germany) and digital images were linearly adjusted in Metaviewer software (Metasystems, GmbH, Altlussheim, Germany). Fiber type and cross-sectional area were quantified using semi-automated image analysis with ImageJ software (National Institutes of Health, Frederick, MD).

### RNA extraction and cDNA synthesis

RNA was extracted from the soleus muscles using a PureLink RNA Mini Kit (Life Technologies) according to the manufacturer's protocol. Total RNA concentration was measured using the NanoDrop 1000 Spectrophotometer (Thermo Scientific, Waltman, MA). Single stranded cDNA was synthesized from 1000 ng of total RNA using a high-capacity cDNA Archive Kit (Applied Biosystems, Warrington, UK).

### Gene expression analysis

Predesigned primer/probe sets for the 13 mitochondrial DNA (mtDNA) encoded subunits of the respiratory complexes I-V, Irs-1, Glut4 and Taqman Universal Mastermix were purchased from Applied Biosystems (supplementary Table 1). ABI 7700 Sequence Detection System (Applied Biosystems) was used for real-time PCR (RT-PCR) to quantify mRNA expression. As a control for between-sample variability, mRNA levels of metabolic and mitochondrial encoded genes were normalized to the geometric mean of the three housekeeping genes glyceraldehyde 3-phosphate dehydrogenase (*Gapdh*), β-2 microglobulin (*B2 m*) and β-actin (*Actb*) (Vandesompele et al., 2002). The relative expression of the gene of interest was calculated using the 2^−ΔΔCT^ method (Livak and Schmittgen, 2001). Results are reported as arbitrary units. Myosin heavy chain (MHC) gene expression was quantified using the LightCycler 480 SYBER Green I Master (Roche Applied Science, Indianapolis, IN). Normalization of MHC genes was performed using the geometric mean of the 3 reference genes: hypoxanthine phosphoribosyltransferase 1 (*Hprt1*), Cyclophilin A (*Ppia*), and *Actb* using the 2^−ΔΔCT^ method. Primer sequences and catalog numbers are provided in supplementary Tables 1, 2.

### Mitochondrial PCR array

cDNA was synthesized from 400 ng of total RNA using the RT2 First Strand Kit (SABioscience, Qiagen, Venlo, Netherlands). The expression of 84 genes that encode for proteins of the ETS complexes were analyzed using the Mitochondrial Energy Metabolism PCR Array profiler (SABioscience). Gene expression was quantified by quantitative RT-PCR on LightCycler 480 SYBR Green I Master (Roche Applied Science, Indianapolis, IN). To control for between-sample variability, mRNA levels were normalized to the geometric mean of a panel of housekeeping genes (60 S acidic ribosomal protein P1 (*Rplp1*), *Hprt1*, and lactate dehydrogenase A (*Ldha*)) using the 2^−ΔΔCT^ method for relative quantification to determine fold change. After normalization, genes which were differently expressed between the CD and HF groups were identified based on the False Discovery Rate calculation with an alpha of *p* < 0.05 (Curran-Everett, 2000). Heat maps were generated using online gene expression software analysis package provided by SABioscience (Frederick, Maryland).

### mtDNA RT-PCR

Genomic DNA was extracted from the soleus muscle using DNA columns from the AllPrep DNA/RNA/miRNA Universal Kit (Qiagen) according to the manufacturer's protocol. Total DNA concentration was measured using the NanoDrop 1000 Spectrophotometer (Thermo Scientific). Two nanograms of total DNA was used in a 10 μl PCR reaction against *Mt-nd1* and *Gapdh* Taqman probes (Applied Biosystems). Mt DNA copy number was estimated as a ratio of mitochondrial-encoded NADH-ubiquinone oxidoreductase chain 1 to GAPDH.

### Immunoblotting

Frozen skeletal muscle was weighed and ~50 mg muscle homogenized using a bead mill homogeniser (OMNI Ruptor, Omni International, Kennesaw, GA) in ice-cold modified RIPA buffer (Millipore #20-188) supplemented with a protease and phosphatase inhibitor cocktail (Halt™ Protease and Phosphatase Inhibitor Cocktail, Thermo Scientific). Homogenates were centrifuged at 14,000 × g for 10 min at 4°C to remove cellular debris, and supernatants were frozen at −80°C until further use. The soluble protein concentration was determined using a BCA protein assay kit, as per the manufacturer's protocol (Pierce, Rockford, IL).

Sample aliquots containing 20 μg of protein were suspended in 1x Laemmli buffer (10% glycerol, 0.5M Tris-HCL, pH 6.8, 1% bromophenol blue, 400 mM dithiothreitol), boiled at 100°C for 5 min and subjected to SDS/PAGE. Proteins were transferred to a pre-soaked PVDF membrane (Bio-Rad, Hercules, CA), incubated with blocking buffer (5% BSA in Tris Buffer Saline with 0.1% Tween 20, (TBST)) for 1 h at room temperature, followed by overnight incubation at 4°C with a commercially available cocktail of primary antibodies against OXPHOS complexes (#110413; 1:1000, Abcam, Cambridge, MA) in blocking buffer under gentle agitation. Membranes were washed five times for 5 min and probed with an anti-rabbit or anti-mouse IgG conjugated to horseradish peroxidase (HRP) secondary antibody in blocking buffer for 1 h at room temperature. Membranes were washed for 25 min in TBST and protein bands were visualized using Amersham ECL Select Western blotting detection reagent (GE Healthcare, Piscataway, NJ). Signals were captured using a Chemidoc™ MP Imaging System (BioRad) and densitometry band analysis was undertaken with ImageJ software. Equal protein loading was determined by staining with Coomassie Brilliant Blue (Thermo Scientific).

### Metabolic enzymatic activities

Enzymatic activities for citrate synthase (CS), lactate dehydrogenase (LDH), 3-hydroxyacyl-CoA dehydrogenase (HADH) and carnitine palmitoyl transferase-1 (CPT1) were determined as previously described (Ihsan et al., 2015). Briefly, tissue was weighed and homogenized in ice-cold homogenization buffer (25 mM TRIS-HCL pH 7.8, 1 mM EDTA, 2 mM MgCl_2_, 50 mM KCl, 0.50% Triton X-100) using a Qiagen TissueLyser II (Qiagen, Dusseldorf, Germany). Homogenates were centrifuged at 14,000 × g for 10 min at 4°C, and the supernatant was frozen at −80°C until further use. All assays were performed using the Molecular Devices Spectramax-340 96-well microplate reading spectrophotometer at 25°C. CS and CPT1 activity were determined by measuring absorbance at 412 nm in 50 mM Tris-HCl (pH 8.0) with 0.2 mM DTNB. CS assays contained 0.1 mM acetyl-coA and 0.25 mM oxaloacetate. CPT1 assays contained 150 mM KCl, 0.1 mM palmitoyl-CoA and 0.25 mM l-carnitine. LDH and HADH activity were determined by measuring absorption at 340 nm. LDH assays contained 100 mM Tris-HCl (pH 7.0), 1 mM EDTA, 2 mM MgCl_2_, 1 mM DTT, 0.15 mM NADH, and 0.15 mM pyruvate. HADH assays contained 50 mM Tris-HCl (pH 7.4), 5.4 mM Acetoacetyl CoA and 6.4 mM NADH. Rate of change of absorbance and path length of each well were determined using SoftMax pro version 3.1.1 (Molecular Devices, Sunnyvale, CA), and enzyme activities were calculated using extinction coefficients of 13.6 mM^−1^cm^−1^ for CS and CPT1, and 6.22 mM^−1^cm^−1^ for LDH and HADH.

### Maximal mitochondrial respiratory complex activities

Enzyme activities for Complex I-IV were determined following the protocols of Spinazzi et al. (2012) adapted to 96-well plate format. All assays were performed using a Molecular Devices Spectramax-340 spectrophotometer (Molecular Devices, Sunnyvale, CA) at 37°C. In brief, analysis for complex I was carried out by measuring the decrease in NADH absorbance at 340 nm in 50 mM potassium phosphate buffer (pH 7.5) with 100 μM NADH, 60 μM Coenzyme Q_1_, 3 mg/ml fatty acid free (FAF)-BSA, and 300 μM KCN. Complex II was analyzed by measuring the reduction of dichlorophenolindopenol (DCPIP) through decreased absorbance at 600 nm at 25 mM potassium phosphate buffer (pH 7.5), 20 mM succinate, 80 μM DCPIP, 50 μM decylubiquinone, 1 mg/ml FAF-BSA, and 300 μM KCN. Complex III activity was analyzed by measuring the reduction of cytochrome c through increased absorbance at 550 nm in 0.025% tween-20 in 25 mM potassium phosphate buffer (pH 7.5), 100 μM decylubiquinol, 75 μM cytochrome C, 500 μM KCN, and 100 μM EDTA. Complex IV was analyzed by measuring the oxidation of cytochrome C through decreased absorption at 550 nm in 50 mM potassium phosphate buffer (pH 7.0), and 50 μM of reduced cytochrome C. Complex I + III linked activity was determined by measuring the reduction of cytochrome C through increased absorbance at 550 nm in 50 mM potassium phosphate buffer (pH 7.5), 200 μM NADH, 50 μM cytochrome C, 1 mg/ml FAF-BSA, and 300 μM KCN. Complex II + III linked activity was determined by measuring the reduction of cytochrome C at 550 nm in 0.5 M potassium phosphate buffer (pH 7.5), 10 mM succinate, 50 μM cytochrome C, and 300 μM KCN. Specificity of Complex I-IV assays was determined by inhibition with rotenone (10 μM), malonate (10 mM), antimycin-a (10 μg/mL), or KCN (300 μM), respectively. Activities were calculated using extinction coefficients (mmol^−1^cm^−1^ (CI ε = 6.2, CII ε = 19.1, CIII ε = 18.5, CIV ε = 18.5, CI + III ε = 18.5, CII + III ε = 13.6). Individual activities were normalized to the activity of citrate synthase (Reisch and Elpeleg, 2007).

### Statistical analysis

Statistical analysis was performed using SigmaPlot for Windows version 12.5 (Systat Software Inc., San Jose, CA). Statistical significance between the CD and HF group was determined using *t*-tests. Data were checked for normality of the residual error distribution and log_10_ transformed where appropriate. Prism software (GraphPad Software Inc., La Jolla, CA) was used to generate graphs. The type I error rate of the mitochondrial gene expression array was controlled for multiple comparisons via the false discovery rate (FDR) approach. Data are shown as means ± SEM. Statistical significance was accepted at *p* < 0.05.

## Results

### Morphometrics and skeletal muscle insulin sensitivity

Maternal characteristics and offspring bodyweights have been reported previously (Pileggi et al., 2015; Segovia et al., 2015). Soleus mass did not differ between groups (Table 1). Skeletal muscle *Irs1* and *Glut4* mRNA expression was decreased in hf offspring (*p* < 0.05; Table 1).

**Table 1:** **Morphometrics, and skeletal muscle enzyme activity**.

	**CD Offspring**	**HF Offspring**	***P*-Value**
Soleus weight (mg)	259.3±13.0	264.38±11.8	0.774
Soleus weight, % body weight	0.40±0.02	0.372±0.02	0.178
IRS-1 gene expression (relative to CD)	1.0±0.09	0.76±0.05^*^	0.039
GLUT4 gene expression (relative to CD)	1.0±0.11	0.69±0.06^*^	0.033
mtDNA/nDNA (relative to CD)	1.0±0.07	0.85±0.06	0.112
**METABOLIC ENZYME ACTIVITY**			
Citrate synthase (nM/min/μg protein)	2566.26±317.90	2891.19±308.25	0.474
Lactate dehydrogenase (nM/min/μg protein)	2261.62±247.69	2308.74±222.31	0.829
Carnitine palmitoyl transferase-1 (nM/min/μg protein)	20.78±1.14	31.07±2.98^*^	0.004
Hydroxyacyl-Coenzyme A dehydrogenase (nM/min/μg protein)	188.60±23.21	214.04±19.27	0.271

Soleus muscle protein was extracted to analyse activity of metabolic enzymes (citrate synthase, lactate dehydrogenase, carnitine palmitoyl transferase 1, and HADH)

* *p < 0.05 CD vs. HF; n = 5–6 litters per group. Data are expressed as means ± SEM, relative to total protein yield*.

### Skeletal muscle fiber type

**Figure 1:**
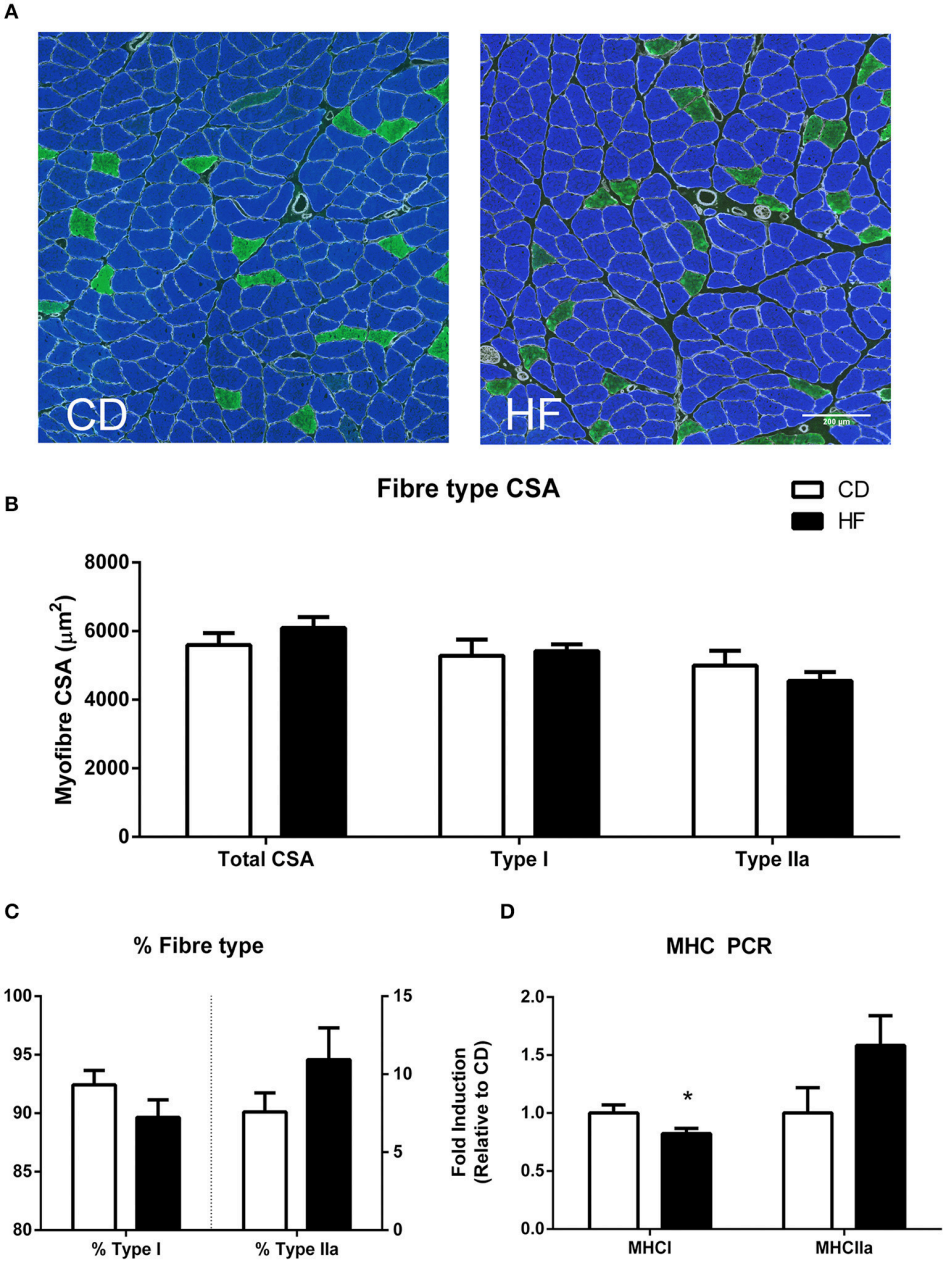
**Skeletal muscle fiber type in adult male offspring. (A)** Soleus representative images. Blue staining represents type I fibers, and green staining type IIa fibers **(B)** soleus myofiber cross sectional area **(C)** percentage fiber type composition **(D)** gene expression of MHC genes. ^*^p < 0.05, CD vs. HF; *n* = 5–6 litters/group. Data are expressed as means ± SEM.

Average muscle fiber cross sectional area, type I fiber cross sectional area and type IIa cross sectional area were not different between groups as examined by immunofluorescence (Figures 1A,B). Fiber-type percentage of both type I and type IIa fibers was not significantly different between groups (Figure 1C). RT-PCR analysis revealed decreased expression of the mRNA encoding the myosin heavy chain type I isoform (*MyH7* gene; *p* < 0.05), with no significant difference in the myosin heavy chain IIa isoform (*Myh2* gene) between groups (Figure 1D).

### Metabolic enzymes

There was no difference in CS, LDH or β-HADH activity between groups (Table 1). However, CPT1 activity was increased in HF offspring (*p* < 0.01; Table 1).

### Mitochondrial transcription factors

**Figure 2:**
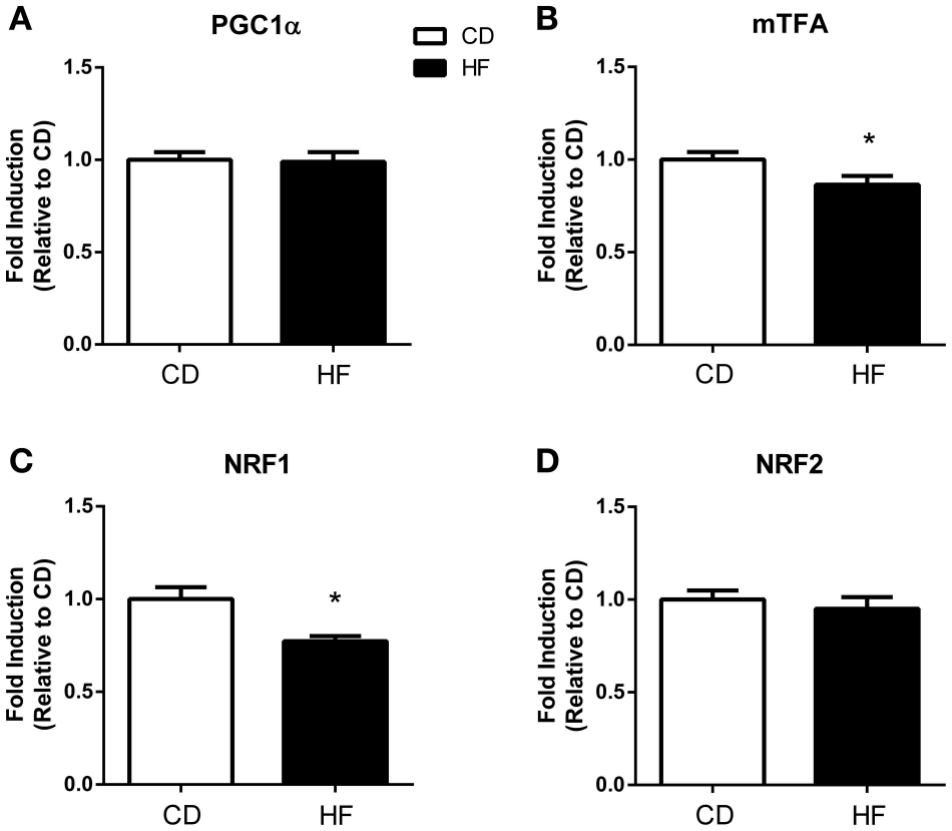
**Skeletal muscle mitochondrial biogenesis in adult male offspring**. Fold induction of mitochondrial biogenesis gene expression in skeletal muscle of 150 days old adult male offspring. **(A)** PGC1α **(B)** mtTFA **(C)** NRF1 **(D)** NRF2. ^*^p < 0.05, CD vs. HF; *n* = 5–6 litters/group. Data are expressed as means ± SEM.

PGC1α and NRF 2 gene expression did not differ between groups (Figures 2A,D, respectively). Expression of both the mitochondrial transcription factor A (mTFA: Figure 1B) and NRF1 were lower in the HF offspring (Figure 1C) (*p* < 0.05 and *p* < 0.01, respectively).

### mtDNA copy number

The ratio of mtDNA/nDNA, determined by mt-ND1/GAPDH, was decreased by 15% in HF offspring, however this was not statistically significant (*p* = 0.112; Table 1).

### Mitochondrial respiratory complex gene expression

**Figure 3:**
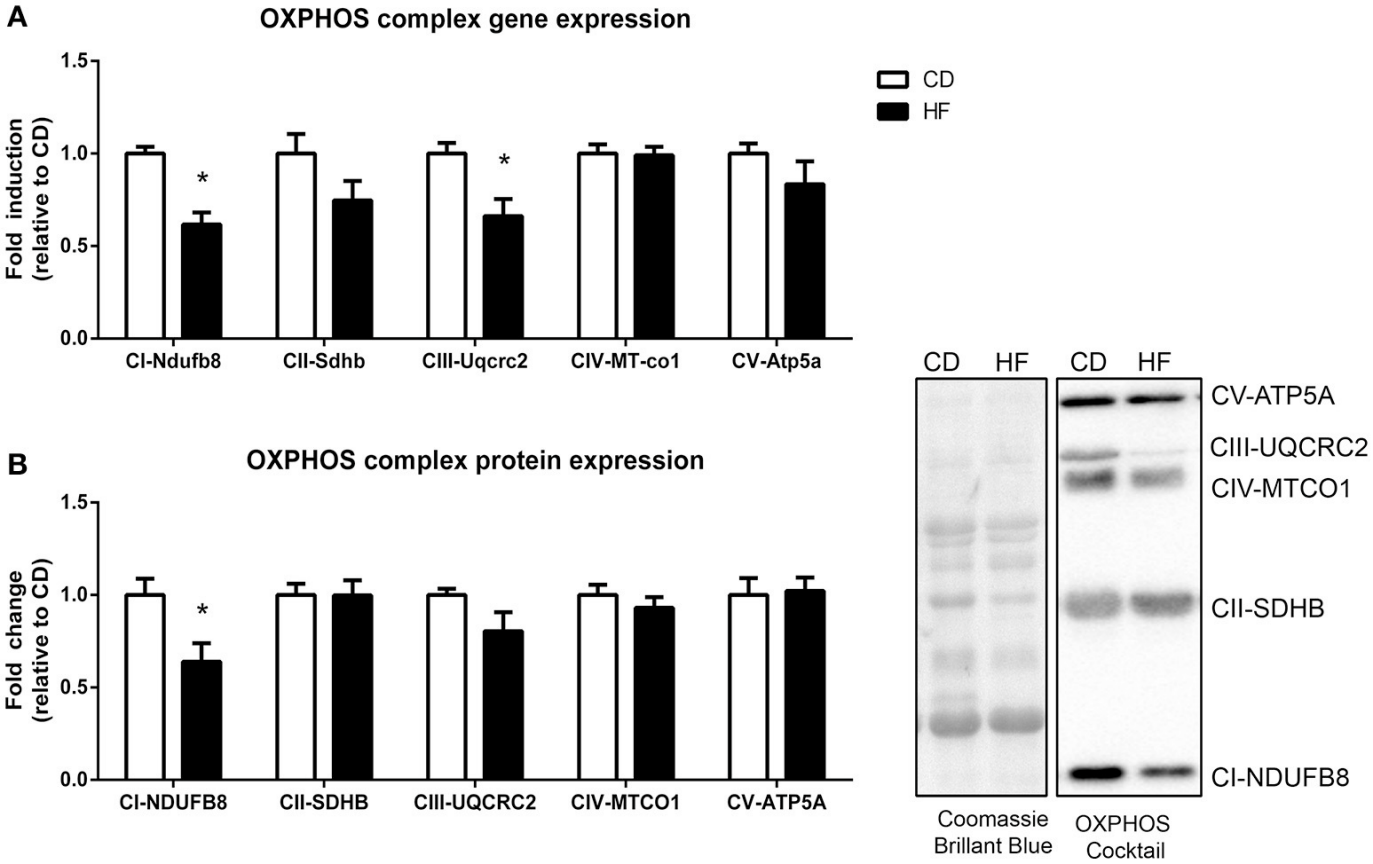
**Skeletal muscle gene and protein expression of mitochondrial complex subunits in adult male offspring**. **(A)** RNA was extracted and RT-PCR array technology was utilized to determine expression of NADH ubiquinone dehydrogenase 1β subcomplex 8 (*Ndfub8*), succinate dehydrogenase iron-sulfur subunit (*Sdhb*), Cytochrome b-c1 complex subunit 2 (*Uqcrc2*), mitochondrial encoded cyctochrome c oxidase I (*Mt-co1*), and ATP synthase α (*Atp5a*) **(B)** Soleus muscle protein was extracted and protein expression of NDUFB8, SDHB, UQCRC2, MT-CO1 and ATP5 was determine by western blot. Representative blots are shown. ^*^p < 0.05, CD vs. HF; *n* = 5–6 litters/group. Data are expressed as means ± SEM.

**Figure 4:**
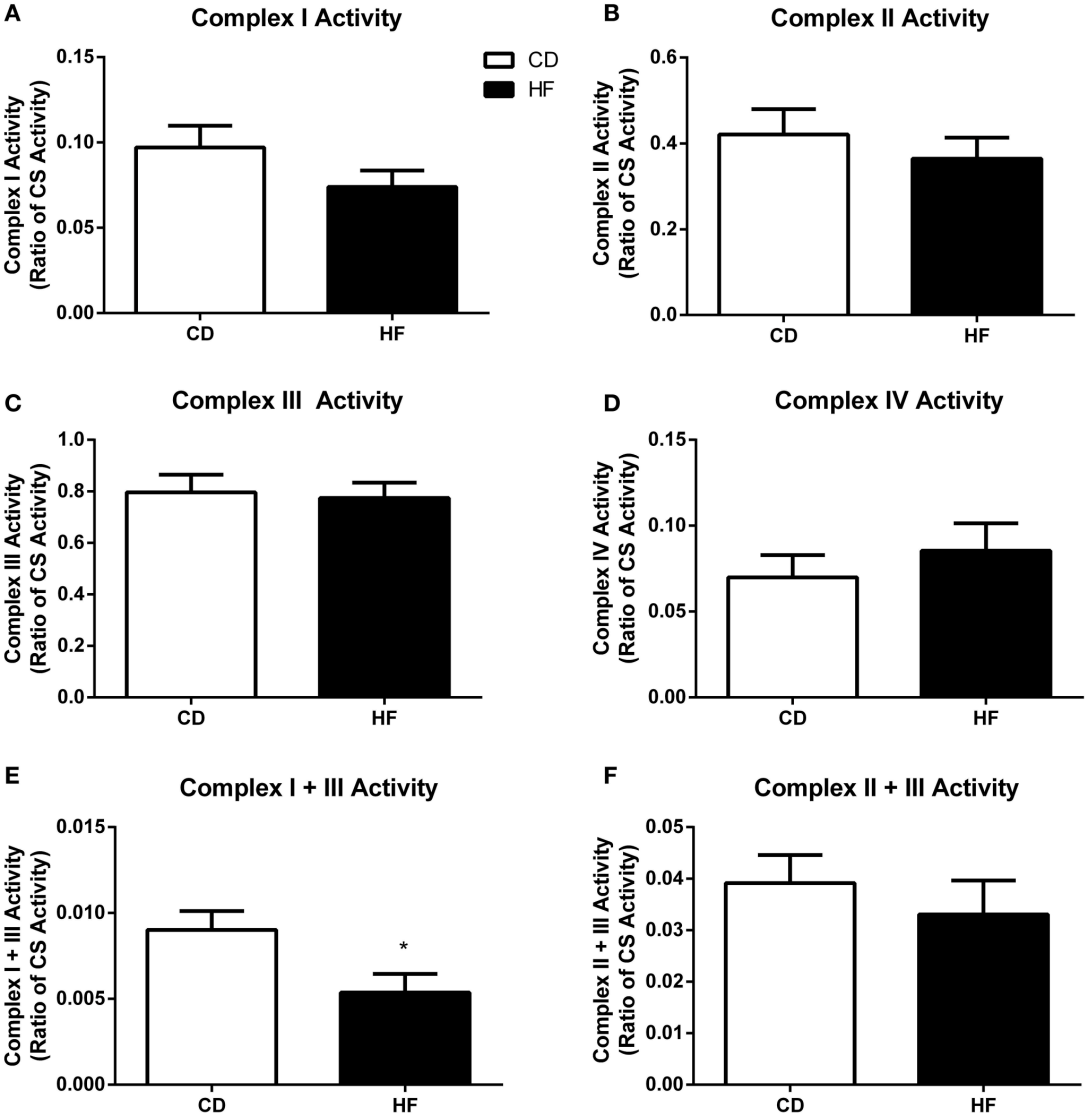
**Skeletal muscle mitochondrial complex activities in adult male offspring**. Soleus muscle protein was extracted and activity of complex I **(A)**, complex II **(B)**, complex III **(C)**, complex IV **(D)**, complex I + III linked **(E)**, and complex II + III **(F)** linked were determined. ^*^p < 0.05, CD vs. HF; *n* = 5–6 litters/group. Data are expressed relative to CS activity ± SEM.

Multiple nuclear genes of the mitochondrial electron transport system complexes, as measured by PCR array, were downregulated in skeletal muscle of HF offspring. In total, 8 genes from complex I, 2 genes from complex II, 2 genes from complex III, 6 genes from complex IV, 7 genes from complex V, and 4 genes encoding for accessory proteins were identified as significantly downregulated in HF offspring after correcting for FDR (*p* < 0.05; Table 2, Figure 3, Supplementary Table 3). Gene expression of both *Ndufb8* and *Uqcrc2* was downregulated in HF offspring (*p* < 0.05), whereas *Sdhb, Mt-co1*, and *Atp5a* expression did not differ between groups (Figure 4A). In contrast, only one mitochondrial encoded complex gene (*Mt-cyb*) differed between groups, being decreased in HF offspring (*p* < 0.05; Supplementary Table 4).

**Table 2:** **RT-PCR array differentially expressed genes**.

**Complex**	**Symbol**	**GeneBank**	**Fold change (Relative to CD)**	***P*-Value**
Complex I (NADH ubiquinone oxidoreductase)	Ndufs7	NM_001008525	−1.98	0.002
	Ndufs3	NM_001106489	−1.8	< 0.001
	Ndufa8	NM_001047862	−1.78	0.001
	Ndufs1	NM_001005550	−1.62	0.009
	Ndufb5	NM_001106426	−1.55	0.012
	Ndufa5	NM_012985	−1.44	0.004
	Ndufb3	NM_001106912	−1.43	0.022
	Ndufb8	NM_001106360	−1.36	0.012
Complex II (Succinate dehydrogenase)	Sdha	NM_130428	−1.8	0.008
	Sdhc	NM_001005534	−1.29	0.013
Complex III (Coenzyme Q- cytochrome C reductase)	Cyc1	NM_001130491	−2.16	< 0.001
	Uqcrc2	NM_001006970	−1.49	0.012
Complex IV (Cytochrome C oxidase)	Cox6a2	NM_012812	−1.65	0.011
	Cox15	NM_001033699	−1.59	0.003
	Cox4i2	NM_053472	−1.57	0.004
	Cox4i1	NM_017202	−1.55	0.009
	Cox7b	NM_182819	−1.51	0.012
	Cox7a2l	NM_001106704	−1.4	0.024
Complex V (ATP Synthase)	Atp6ap1	NM_031785	−1.87	0.022
	Atp5b	NM_134364	−1.66	0.006
	Atp5g2	NM_133556	−1.55	0.004
	Atp5l	NM_212516	−1.44	0.024
	Atp5i	NM_080481	−1.38	0.006
	Atp5d	NM_139106	−1.38	0.020
	Atp5h	NM_019383	−1.35	0.001
Accessory Proteins	Ucp2	NM_019354	−3.1	0.001
	Ucp3	NM_013167	−1.71	0.009
	Lhpp	NM_001009706	−1.65	0.010
	Ppa1	NM_001100834	−1.48	0.016

*RT-PCR array analysis identified 29 genes that were differentially expressed in HF offspring. Of the 29 genes that were down regulated, 8 genes encode for complex I (NADH ubiquinone oxioreductase), 2 encode for complex II (succinate dehydrogenase), 2 encode for complex III (Coenzyme Q-cytochrome C reductase), 6 encode for complex IV (cytochrome c oxidase), 7 encode for complex V (ATPsynthase), and 4 accessory genes that encode for proteins involved in oxidative metabolism. Colors reflect fold change intensity*.

### Skeletal muscle OXPHOS complex protein expression

Protein expression of a single subunit of complex I (NADH dehydrogenase (NDUFB8)) was decreased (*p* < 0.05) in HF offspring (Figure 4B). The apparent tendency for Complex III protein expression to be decreased [Q-cytochrome c oxidoreductase (QCRC2)] in HF offspring was not significant (*p* = 0.12). Protein expression of complex II [succinate dehydrogenase (SDHB)], complex IV [Cytochrome C oxidase (MTCO1)] and complex V [ATP synthase (ATP5A)] did not differ between groups (Figure 4B).

### Maximal enzymatic activity of mitochondrial respiratory complexes

There was no difference in activity of complex I (Figure 4A), complex II (Figure 4B), complex III (Figure 4C) complex IV (Figure 4D), and complex II + III (Figure 4F) linked between groups. In contrast, complex I + III linked activity was lower in HF offspring (*p* < 0.05; Figure 4E).

## Discussion

This study demonstrates that a maternal diet high in fat had a significant influence on the regulation, expression, and function of the mitochondrial ETS respiratory complexes in offspring skeletal muscle. Specifically, this present study has identified that the mitochondrial transcription factors NRF1 and mtTFA were decreased, accompanied by a coordinated decline in gene expression of specific mitochondrial ETS complex subunits in the skeletal muscle of offspring born to HF-fed dams. Furthermore, the decrease in gene expression of the NDUFB8 subunit from complex I and UQCRC2 subunit from complex III prompted declines in protein expression of these subunits, which was further complemented by complex I and III linked functional capacity. This study highlights the specific molecular changes that define the impaired mitochondrial phenotype in skeletal muscle from offspring born to mothers fed a HF diet. Taken together, our data suggest that increased maternal fat intake during pregnancy can program mitochondrial dysfunction in skeletal muscle which may have an important role in defining lifelong metabolic health of the offspring.

Deficiencies in mitochondrial content and function are thought to play a causative role in the post-natal development of insulin resistance and associated metabolic diseases (Ritov et al., 2005). Citrate synthase activity, a mitochondrial matrix enzyme, is strongly correlated with mitochondrial content in skeletal muscle (Larsen et al., 2012). The present study found no significant difference in CS activity, indicating that mitochondrial density is not altered in response to a maternal HF diet. Likewise, the absence of differences in LDH and β-HADH activity suggests that glycolytic activity and β-oxidation of fatty acids are preserved in the HF offspring. However, CPT1 activity was increased in HF offspring. CPT1 regulates mitochondrial fatty acid oxidation in muscle by controlling the movement of fatty acids to the intermembrane space of mitochondria (Bonen et al., 1999; Bezaire et al., 2006; Holloway et al., 2006). Increased CPT1 activity suggests that HF offspring have the capacity to oxidize more fat compared to controls. This is consistent with data from post-natal HF diet studies demonstrating an adaptation in CPT1 activity (Bruce et al., 2009b) leading to increased uptake and utilization of fatty acids (Turner et al., 2007). Thus, increased activity of CPT1 in HF offspring suggests there is a capacity for altered skeletal muscle substrate utilization of fatty acids during energy metabolism.

Altered expression of upstream factors essential for mitochondrial transcription and translation of genes that encode mitochondrial proteins can propagate mitochondrial dysfunction. PGC1α is considered the master-regulator of mitochondrial biogenesis by acting as a transcriptional co-activator of NRFs and mtTFA, which, in turn, induce coordinated expression of genes involved in mitochondrial oxidative metabolism (Virbasius and Scarpulla, 1994; Scarpulla, 2002). PGC1α (Mootha et al., 2003) and NRF1 (Patti et al., 2003) were downregulated in muscle tissue from humans exhibiting insulin resistance and T2D subjects, with a corresponding decrease in downstream OXPHOS target genes. Despite the lack of a difference in PGC1α mRNA expression between groups presented in this study, a recent study demonstrated hypermethylation of the PGC1α promoter region in offspring born to HF dams (Laker et al., 2014), suggesting epigenetic control of PGC1α expression in response to a maternal HF diet. In contrast, the present study found decreased expression of NRF1 and mtTFA in HF offspring, which should also result in similar down regulation of oxidative metabolism. Many nuclear encoded genes involved in oxidative metabolism, including genes that comprise the ETS complexes, are regulated by NRF1-dependent transcription (Scarpulla, 2002). In this regard, there is a critical role for NRF1 in the downstream transcriptional regulation of mitochondrial proteins.

Due to the large number of genes encoding components of five ETS respiratory complexes, PCR array technology was utilized to identify differentially expressed genes. Multiple nuclear-encoded mitochondrial genes for subunits of each ETS complex were downregulated in HF offspring. Decreased expression of the mitochondrial ETS complexes suggests defective oxidative energy metabolism in HF offspring correlating with decreased expression of GLUT4 and IRS1 in skeletal muscle. Previous short-term intervention studies have revealed that high post-natal dietary fat intake markedly down-regulates several ETS respiratory complex genes in skeletal muscle following both 3 days of an isoenergetic HF diet in young healthy human males and a 21 day HF intervention in male mice (Sparks et al., 2005). Of the differentially expressed genes identified in these studies, the complex I (Ndufb5, Ndufb3, Ndufs1, Ndufv1) and III genes (Cyc1) were reportedly downregulated. This is consistent with the findings presented in this current study, suggesting similar mechanisms for maternal HF in the progression of mitochondrial dysfunction as with post-natal HF feeding. Importantly, studies on human T2D subjects and subjects with a family history of T2D have reported similar decreases in gene expression of the mitochondrial ETS respiratory complexes subunits (Mootha et al., 2003; Patti et al., 2003). Of note, the similarity between the results reported here and in previous studies on short term HF diets and T2D subjects, suggests that altering maternal diet can elicit mitochondrial perturbations in offspring muscle, and these are consistent with metabolic diseases. Moreover, while expression of the mitochondrially-encoded subunits were largely unaffected by the decrease in mtTFA, mtDNA copy number was decreased in HF offspring. These findings are in-line with reports showing that mammalian mtTFA can regulate mtDNA copy number independent of changes in mitochondrial proteins (Ekstrand et al., 2004).

Analyses of protein expression and maximal enzymatic activity of the mitochondrial ETS respiratory complexes was undertaken to assess the functional impact of the observed alterations in the expression of OXPHOS genes. HF offspring displayed significantly lower levels of protein expression of complex I (NADH dehydrogenase, NDUFB8). Notably, the changes in protein expression for subunits from each of the ETS respiratory complexes are consistent with changes in gene expression established in the PCR array. Specifically, both Ndufb8 and Uqcrc2 gene and protein expression were downregulated in HF offspring compared to controls, whereas gene and protein expression of complex II (Sdhb), complex IV (Mt-co1), and complex V (Atp-5a) did not differ between groups. The decreases in protein expression of complex I and complex III did not cause a downregulation in individual complex activity. However, emerging evidence suggests that electron flux and proton pumping capacity of ETS complexes may be in part determined by ultrastructural assembly into supercomplexes (Dudkina et al., 2010; Lapuente-Brun et al., 2013). These supercomplexes consist of combinations of complexes I, III and IV which optimizes electron transfer by decreasing diffusion distance of electron carriers between complexes. Therefore, despite no difference in activity of complexes I, II, III, or IV, overall ETS function could be impaired due to the alterations in formation and function of supercomplexes (Schägger, 2001; Dudkina et al., 2010). Consequently, the observed decrease in maximal catalytic complex I and III linked activity in HF offspring may indicate an impairment in the efficiency of electron transfer. This may in turn affect proton pumping capacity and either impair ATP generation or result in a compensatory mechanism to maintain membrane potential for ATP generation (Acín-Pérez et al., 2004; Dudkina et al., 2008). Thus, the observed decrease in maximal complex I and III linked activity indicates that a maternal HF diet elicits functional defects in mitochondrial oxidative capacity in offspring which may contribute to the pathophysiology of metabolic disease.

In conclusion, the present study demonstrates that a maternal diet high in saturated fat can negatively impact specific molecular components involved in defining offspring skeletal muscle mitochondrial function. Skeletal muscle from HF offspring exhibited decreased expression of NRF1 and mTFA with a coordinated suppression of specific downstream mitochondrial ETS complex genes. These changes in gene expression translated into a selective decrease in mitochondrial protein expression and activity of ETS complexes I and III. Taken together, these data indicate that increased maternal fat intake during pregnancy and lactation may program mitochondrial dysfunction that propagates the development of metabolic disease in adult offspring. We postulate that alterations in substrate utilization and energy demands may contribute to poor metabolic outcomes in offspring. While this study provides evidence of the specific mitochondrial molecular mechanisms in skeletal muscle affected by a maternal HF diet, further studies utilizing mitochondrial glucose and lipid-base substrates are required to understand the functional significance of these alterations, and the clinical implications to human health.

## Author contributions

Conceived and designed the experiments: CAP, MHV, CPH and CMR. Performed the experiments: CAP, CMR, CPH, SAS, BRD, XDZ, and CG. Analyzed data: CAP, CMR, BRD, and CPH. Wrote the paper: CAP. Critically evaluated the paper: CG, CMR, JFM, CPH, MHV, AJRH, MPGB, and DCS.

## Funding

The project was funded by Gravida, National Centre for Growth and Development; MP16 Rat Programme Part 1 with additional funding for analysis through AgResearch Limited (contracts A19079 and A21246) and the University of Auckland, the Kelliher Charitable Trust, Faculty Research Development Fund (FRDF). CMR was supported by an Auckland Medical Research Foundation (AMRF) David and Cassie Anderson Fellowship. CAP was supported by PhD scholarships from AgResearch Limited and the Agnes Paykel Trust.

### Conflict of interest statement

The authors declare that the research was conducted in the absence of any commercial or financial relationships that could be construed as a potential conflict of interest.

## Abbreviations

CPT1: Carnitine palmitoyl transferase 1
CS: Citrate synthase
ETS: Electron transport system
HF: High fat
LDH: Lactate dehydrogenase
mtTFA: Mitochondrial transcription factor A
NRF: Nuclear respiratory factor
OXPHOS: Oxidative phosphorylation
PGC1α: proliferator-activated receptor γ coactivator α
T2D: Type II diabetes.
