# Supplementary material for: Maternal High Fat Diet Alters Skeletal Muscle Mitochondrial Catalytic Activity in Adult Male Rat Offspring
Source: Front Physiol. 2016 Nov 18;7:546. doi: 10.3389/fphys.2016.00546 (PMC5114294; doi:10.3389/fphys.2016.00546)
Supplement: Supplementary file 1 [file Table1.DOCX]

| Gene Name | GeneBank | Symbol | Catalogue Number |
| --- | --- | --- | --- |
| mitochondrially encoded NADH dehydrogenase 1 | NC_001665.ND1.0 | Mt-ND1 | Rn03296764_s1 |
| mitochondrially encoded NADH dehydrogenase 2 | NC_001665.ND2.0 | Mt-ND2 | Rn03296765_s1 |
| mitochondrially encoded NADH dehydrogenase 3 | NC_001665.ND3.0 | Mt-ND3 | Rn03296825_s1 |
| mitochondrially encoded NADH dehydrogenase 4 | NC_001665.ND4.0 | Mt-ND4 | Rn03296781_s1 |
| mitochondrially encoded NADH 4L | NC_001665.ND4L.0 | Mt-ND4L | Rn03296792_s1 |
| mitochondrially encoded NADH dehydrogenase 5 | NC_001665.ND5.0 | Mt-ND5 | Rn03296799_s1 |
| mitochondrially encoded NADH dehydrogenase 6 | NC_001665.ND6.0 | Mt-ND6 | Rn03296815_s1 |
| mitochondrially encoded cytochrome b | NC_001665.CYTB.0 | Mt-Cyb | Rn03296746_s1 |
| mitochondrially encoded cytochrome c oxidase I | NC_001665.COX1.0 | Mt-Co1 | Rn03296721_s1 |
| mitochondrially encoded cytochrome c oxidase II | NC_001665.COX2.0 | Mt-Co2 | Rn03296737_s1 |
| mitochondrially encoded cytochrome c oxidase III | NC_001665.COX3.0 | Mt-Co3 | Rn03296820_s1 |
| mitochondrially encoded ATP synthase 6 | NC_001665.ATP6.0 | Mt-ATP6 | Rn03296710_s1 |
| mitochondrially encoded ATP synthase 8 | NC_001665.ATP8.0 | Mt-ATP8 | Rn03296716_s1 |
| insulin receptor substrate 1 | NM_012969.1 | IRS-1 | Rn02132493_s1 |
| solute carrier family 2 (facilitated glucose transporter), member 4 | NM_012751.1 | Slc2a4 | Rn01752377_m1 |
| actin, beta | NM_031144.3 | Actb | Rn00667869_m1 |
| glyceraldehyde-3-phosphate dehydrogenase | NM_017008.4 | Gapdh | Rn01775763_g1 |
| beta-2 microglobulin | NM_012512.2 | B2m | Rn00560865_m1 |

**Supplementary Table 1** Catalogue numbers for the predesigned primer/probe sets used for RT-PCR analysis of the 13 mitochondrial DNA (mtDNA) encoded subunits of the respiratory complexes I-V, metabolic and housekeeping genes.

| RefSeq | Gene name | Symbol | Forward primer | Reverse Primer |
| --- | --- | --- | --- | --- |
| NM_017240.2 | Myosin, heavy chain 7, cardiac muscle, beta (MHCI), mRNA | *Myh7* | AACAGGCCAACACCAACCTG | CTACTCTTCATTCAGGCCCTTGG |
| NM_001135157.1 | Myosin, heavy chain 2, skeletal muscle, adult (MHCIIa), mRNA | *Myh2* | GCCGCGAGGTTCACACTAAA | TTTGTGCCTCTCTTCGGTCA |
| NM_031144.3 | Actin, beta, mRNA | *Actb* | CCGCGAGTACAACCTTCTTG | CATCCATGGCGAACTGGTGG |
| NM_012583.2 | Hypoxanthine phosphoribosyltransferase 1, mRNA | *Hprt1* | GTCAAGCAGTACAGCCCCAA | CAAATCCAACAAAGTCTGGCCT |
| NM_017101.1 | Peptidylprolyl isomerase A (cyclophilin A), mRNA | *Ppia* | TGTTCTTCGACATCACGGCT | GCACGAAAGTTTTCTGCTGTCT |

**Supplementary Table 2** Primer pairs for MHC genes

| Unigene | GeneBank | Symbol | Description | P Value | Fold change (Relative to CD) | False Discovery Rate Correction |
| --- | --- | --- | --- | --- | --- | --- |
| Rn.1261 | NM_001106489 | Ndufs3 | NADH dehydrogenase (ubiquinone) Fe-S protein 3 | 0.000049 | -1.8 | TRUE |
| Rn.1413 | NM_001130491 | Cyc1 | Cytochrome c-1 | 0.000124 | -2.16 | TRUE |
| Rn.13333 | NM_019354 | Ucp2 | Uncoupling protein 2 (mitochondrial, proton carrier) | 0.000557 | -3.1 | TRUE |
| Rn.1128 | NM_001047862 | Ndufa8 | NADH dehydrogenase (ubiquinone) 1 alpha subcomplex, 8 | 0.000926 | -1.78 | TRUE |
| Rn.224479 | NM_019383 | Atp5h | ATP synthase, H+ transporting, mitochondrial F0 complex, subunit d | 0.001091 | -1.35 | TRUE |
| Rn.2855 | NM_001008525 | Ndufs7 | NADH dehydrogenase (ubiquinone) Fe-S protein 7 | 0.002381 | -1.98 | TRUE |
| Rn.1309 | NM_001033699 | Cox15 | COX15 homolog, cytochrome c oxidase assembly protein (yeast) | 0.003012 | -1.59 | TRUE |
| Rn.127811 | NM_133556 | Atp5g2 | ATP synthase, H+ transporting, mitochondrial F0 complex, subunit C2 (subunit 9) | 0.003697 | -1.55 | TRUE |
| Rn.100240 | NM_012985 | Ndufa5 | NADH dehydrogenase (ubiquinone) 1 alpha subcomplex 5 | 0.003992 | -1.44 | TRUE |
| Rn.7214 | NM_053472 | Cox4i2 | Cytochrome c oxidase subunit IV isoform 2 | 0.004010 | -1.57 | TRUE |
| Rn.66347 | NM_080481 | Atp5i | ATP synthase, H+ transporting, mitochondrial F0 complex, subunit e | 0.005767 | -1.38 | TRUE |
| Rn.92965 | NM_134364 | Atp5b | ATP synthase, H+ transporting, mitochondrial F1 complex, beta polypeptide | 0.005985 | -1.66 | TRUE |
| Rn.228607 | NM_130428 | Sdha | Succinate dehydrogenase complex, subunit A, flavoprotein (Fp) | 0.008392 | -1.8 | TRUE |
| Rn.9902 | NM_013167 | Ucp3 | Uncoupling protein 3 (mitochondrial, proton carrier) | 0.008766 | -1.71 | TRUE |
| Rn.2528 | NM_017202 | Cox4i1 | Cytochrome c oxidase subunit IV isoform 1 | 0.008840 | -1.55 | TRUE |
| Rn.1467 | NM_001005550 | Ndufs1 | NADH dehydrogenase (ubiquinone) Fe-S protein 1 | 0.008862 | -1.62 | TRUE |
| Rn.15275 | NM_001009706 | Lhpp | Phospholysine phosphohistidine inorganic pyrophosphate phosphatase | 0.010126 | -1.65 | TRUE |
| Rn.5119 | NM_012812 | Cox6a2 | Cytochrome c oxidase subunit VIa polypeptide 2 | 0.011458 | -1.65 | TRUE |
| Rn.2334 | NM_001006970 | Uqcrc2 | Ubiquinol cytochrome c reductase core protein 2 | 0.011517 | -1.49 | TRUE |
| Rn.2026 | NM_182819 | Cox7b | Cytochrome c oxidase subunit VIIb | 0.011903 | -1.51 | TRUE |
| Rn.3367 | NM_001106426 | Ndufb5 | NADH dehydrogenase (ubiquinone) 1 beta subcomplex, 5 | 0.011907 | -1.55 | TRUE |
| Rn.3383 | NM_001106360 | Ndufb8 | NADH dehydrogenase (ubiquinone) 1 beta subcomplex 8 | 0.012417 | -1.36 | TRUE |
| Rn.1698 | NM_001005534 | Sdhc | Succinate dehydrogenase complex, subunit C, integral membrane protein | 0.013383 | -1.29 | TRUE |
| Rn.106916 | NM_001100834 | Ppa1 | Pyrophosphatase (inorganic) 1 | 0.015662 | -1.48 | TRUE |
| Rn.3879 | NM_139106 | Atp5d | ATP synthase, H+ transporting, mitochondrial F1 complex, delta subunit | 0.020147 | -1.38 | TRUE |
| Rn.17057 | NM_001106912 | Ndufb3 | NADH dehydrogenase (ubiquinone) 1 beta subcomplex 3 | 0.021681 | -1.43 | TRUE |
| Rn.93045 | NM_031785 | Atp6ap1 | ATPase, H+ transporting, lysosomal accessory protein 1 | 0.022496 | -1.87 | TRUE |
| Rn.107458 | NM_212516 | Atp5l | ATP synthase, H+ transporting, mitochondrial F0 complex, subunit G | 0.023826 | -1.44 | TRUE |
| Rn.3907 | NM_001106704 | Cox7a2l | Cytochrome c oxidase subunit VIIa polypeptide 2 like | 0.023989 | -1.4 | TRUE |
| Rn.880 | NM_012814 | Cox6a1 | Cytochrome c oxidase, subunit VIa, polypeptide 1 | 0.035597 | -1.57 | FALSE |
| Rn.2270 | NM_134345 | Cox8a | Cytochrome c oxidase subunit VIIIa | 0.036558 | -1.45 | FALSE |
| Rn.15293 | NM_001007666 | Bcs1l | BCS1-like (yeast) | 0.038163 | -1.46 | FALSE |
| Rn.19207 | NM_053540 | Cox17 | COX17 cytochrome c oxidase assembly homolog (S. cerevisiae) | 0.042539 | -1.68 | FALSE |
| Rn.4013 | NM_001130505 | Ndufa6 | NADH dehydrogenase (ubiquinone) 1 alpha subcomplex, 6 (B14) | 0.052571 | -1.22 |  |
| Rn.18013 | NM_001108624 | Ndufb2 | NADH dehydrogenase (ubiquinone) 1 beta subcomplex, 2 | 0.055001 | -1.22 |  |
| Rn.3159 | NM_001006972 | Ndufv1 | NADH dehydrogenase (ubiquinone) flavoprotein 1 | 0.068456 | -1.34 |  |
| Rn.3631 | NM_133418 | Slc25a10 | Solute carrier family 25 (mitochondrial carrier; dicarboxylate transporter), member 10 | 0.070314 | -1.38 |  |
| Rn.3373 | NM_001106322 | Ndufs8 | NADH dehydrogenase (ubiquinone) Fe-S protein 8 | 0.071203 | -1.64 |  |
| Rn.2180 | NM_053756 | Atp5g3 | ATP synthase, H+ transporting, mitochondrial F0 complex, subunit C3 (subunit 9) | 0.075970 | 1.38 |  |
| Rn.63959 | NM_053825 | Atp5c1 | ATP synthase, H+ transporting, mitochondrial F1 complex, gamma polypeptide 1 | 0.082616 | -1.54 |  |
| Rn.797 | NM_001106772 | Ndufa7 | NADH dehydrogenase (ubiquinone) 1 alpha subcomplex, 7 | 0.083371 | -1.63 |  |
| Rn.7401 | NM_001009480 | Uqcrh | Ubiquinol-cytochrome c reductase hinge protein | 0.092443 | -1.22 |  |
| Rn.3128 | NM_001013157 | Nnt | Nicotinamide nucleotide transhydrogenase | 0.093653 | -1.36 |  |
| Rn.1318 | NM_001106294 | Ndufab1 | NADH dehydrogenase (ubiquinone) 1, alpha/beta subcomplex, 1 | 0.122858 | -1.45 |  |
| Rn.74313 | NM_172068 | Surf1 | Surfeit 1 | 0.126540 | -1.39 |  |
| Rn.3902 | NM_001100539 | Sdhb | Succinate dehydrogenase complex, subunit B, iron sulfur (Ip) | 0.126801 | -1.33 |  |
| Rn.104528 | NM_001106646 | Ndufb6 | NADH dehydrogenase (ubiquinone) 1 beta subcomplex, 6 | 0.134476 | -1.44 |  |
| Rn.28882 | NM_031064 | Ndufv2 | NADH dehydrogenase (ubiquinone) flavoprotein 2 | 0.160321 | -1.19 |  |
| Rn.145217 | NM_001014199 | Atp6v1c2 | ATPase, H+ transporting, lysosomal V1 subunit C2 | 0.163304 | -1.45 |  |
| Rn.11077 | NM_145783 | Cox5a | Cytochrome c oxidase, subunit Va | 0.185303 | -1.19 |  |
| Rn.169414 | NM_199495 | Ndufa10 | NADH dehydrogenase (ubiquinone) 1 alpha subcomplex 10 | 0.206807 | -1.2 |  |
| Rn.204067 | NM_053775 | Atp6v0a2 | ATPase, H+ transporting, lysosomal V0 subunit A2 | 0.215281 | -1.22 |  |
| Rn.99666 | NM_001011907 | Ndufs2 | NADH dehydrogenase (ubiquinone) Fe-S protein 2 | 0.216499 | -1.25 |  |
| Rn.40255 | NM_023093 | Atp5a1 | ATP synthase, H+ transporting, mitochondrial F1 complex, alpha subunit 1, cardiac muscle | 0.223824 | -1.29 |  |
| Rn.154403 | NM_019223 | Ndufs6 | NADH dehydrogenase (ubiquinone) Fe-S protein 6 | 0.237974 | -1.19 |  |
| Rn.3040 | NM_198788 | Sdhd | Succinate dehydrogenase complex, subunit D, integral membrane protein | 0.250436 | -1.13 |  |
| Rn.5790 | NM_053602 | Atp5j | ATP synthase, H+ transporting, mitochondrial F0 complex, subunit F6 | 0.283602 | -1.24 |  |
| Rn.2603 | NM_001008888 | Uqcrfs1 | Ubiquinol-cytochrome c reductase, Rieske iron-sulfur polypeptide 1 | 0.290520 | -1.14 |  |
| Rn.163331 | NM_001047880 | Slc25a15 | Solute carrier family 25 (mitochondrial carrier; ornithine transporter) member 15 | 0.301086 | -1.15 |  |
| Rn.3472 | NM_001025134 | Uqcrq | Ubiquinol-cytochrome c reductase, complex III subunit VII | 0.321746 | -1.21 |  |
| Rn.14800 | NM_001105991 | Atp6v1g3 | ATPase, H+ transporting, lysosomal V1 subunit G3 | 0.350013 | 1.14 |  |
| Rn.1817 | NM_138883 | Atp5o | ATP synthase, H+ transporting, mitochondrial F1 complex, O subunit | 0.417160 | -1.18 |  |
| Rn.3289 | NM_053965 | Slc25a20 | Solute carrier family 25 (carnitine/acylcarnitine translocase), member 20 | 0.421495 | -1.08 |  |
| Rn.3689 | NM_134365 | Atp5f1 | ATP synthase, H+ transporting, mitochondrial F0 complex, subunit B1 | 0.433444 | -1.19 |  |
| Rn.9858 | NM_133517 | Atp12a | ATPase, H+/K+ transporting, nongastric, alpha polypeptide | 0.440690 | 1.12 |  |
| Rn.83540 | NM_001011972 | Atp6v0d2 | ATPase, H+ transporting, lysosomal V0 subunit D2 | 0.440690 | 1.12 |  |
| Rn.98192 | NM_183055 | Cox8c | Cytochrome c oxidase, subunit VIIIc | 0.440690 | 1.12 |  |
| Rn.3428 | NM_001004250 | Uqcrc1 | Ubiquinol-cytochrome c reductase core protein I | 0.455032 | -1.12 |  |
| Rn.22045 | NM_001127294 | Ndufb9 | NADH dehydrogenase (ubiquinone) 1 beta subcomplex, 9 | 0.518439 | -1 |  |
| Rn.1745 | NM_022503 | Cox7a2 | Cytochrome c oxidase subunit VIIa polypeptide 2 | 0.551033 | 1.05 |  |
| Rn.161896 | NM_001108979 | Atp6v1e2 | ATPase, H transporting, lysosomal V1 subunit E2 | 0.605902 | 1.01 |  |
| Rn.107963 | NM_001100752 | Ndufa9 | NADH dehydrogenase (ubiquinone) 1 alpha subcomplex, 9 | 0.630862 | 2.46 |  |
| Rn.105421 | NM_001108442 | Ndufb7 | NADH dehydrogenase (ubiquinone) 1 beta subcomplex, 7 | 0.649571 | 1.03 |  |
| Rn.6686 | NM_053586 | Cox5b | Cytochrome c oxidase subunit Vb | 0.665169 | 1.01 |  |
| Rn.846 | NM_019360 | Cox6c | Cytochrome c oxidase, subunit VIc | 0.668159 | -1.08 |  |
| Rn.62471 | NM_001009290 | Ndufc2 | NADH dehydrogenase (ubiquinone) 1, subcomplex unknown, 2 | 0.755449 | -1.03 |  |
| Rn.214529 | NM_012509 | Atp4a | ATPase, H+/K+ exchanging, alpha polypeptide | 0.765671 | 1.02 |  |
| Rn.10033 | NM_012510 | Atp4b | ATPase, H+/K+ exchanging, beta polypeptide | 0.776081 | 1.01 |  |
| Rn.19077 | NM_001106153 | Ndufa2 | NADH dehydrogenase (ubiquinone) 1 alpha subcomplex, 2 | 0.777066 | 1.8 |  |
| Rn.96380 | NM_212517 | Ndufa11 | NADH dehydrogenase (ubiquinone) 1 alpha subcomplex 11 | 0.783488 | -1.02 |  |
| Rn.203141 | NM_001025146 | Ndufs4 | NADH dehydrogenase (ubiquinone) Fe-S protein 4 | 0.866242 | 1.04 |  |
| Rn.10281 | NM_012682 | Ucp1 | Uncoupling protein 1 (mitochondrial, proton carrier) | 0.923367 | -1.02 |  |
| Rn.105517 | NM_001127553 | Uqcrb | Ubiquinol-cytochrome c reductase binding protein | 0.967654 | -1.03 |  |
| Rn.3640 | NM_001108813 | Ndufa1 | NADH dehydrogenase (ubiquinone) 1 alpha subcomplex, 1 | 0.992159 | 4.77 |  |

**Supplementary Table 3** Fold change and accession numbers for the normalised expression of 84 genes encoding the nuclear subunits of the mitochondrial respiratory complexes in soleus muscle of 150 day old adult male offspring

| Complex | Mitochondrial encoded subunit | Fold change (relative to CD) | P Value |
| --- | --- | --- | --- |
| CI (NADH ubiquinone oxidoreductase) | NADH dehydrogenase 1 (mt-ND1) | 1.06±0.11 | 0.695 |
|  | NADH dehydrogenase 2 (mt-ND2) | 0.97±0.14 | 0.886 |
|  | NADH dehydrogenase 3 (mt-ND3) | 0.97±0.12 | 0.872 |
|  | NADH dehydrogenase 4  (mt-ND4) | 1.05±0.08 | 0.720 |
|  | NADH 4L (mt-ND4L) | 1.32±0.13 | 0.076 |
|  | NADH dehydrogenase 5  (mt-ND5) | 1.05±0.072 | 0.614 |
|  | NADH dehydrogenase 6 (mt-ND6) | 1.12±0.83 | 0.258 |
| CIII (Coenzyme Q-cytochrome C reductase) | Cytochrome b (mt-Cyb) | 0.84±0.025* | 0.039 |
| CIV (Cytochrome C oxidase) | Cytochrome c oxidase I (mt-COI) | 0.99±0.046 | 0.444 |
|  | Cytochrome c oxidase II (mt-COII) | 0.95±0.078 | 0.403 |
|  | Cytochrome c oxidase III (mt-COIII) | 1.10±0.11 | 0.407 |
| CV (ATP synthase) | ATP synthase 6 (mt-ATP6) | 1.02±0.09 | 0.403 |
|  | ATP synthase 8 (mt-ATP8) | 1.13±0.069 | 0.116 |

**Supplementary Table 4** Gene expression the 13 subunits of the ETS complexes that are mitochondrial encoded in muscle of 150 day old adult male offspring
